# A Single *Vibrionales* 16S rRNA Oligotype Dominates the Intestinal Microbiome in Two Geographically Separated Atlantic cod Populations

**DOI:** 10.3389/fmicb.2018.01561

**Published:** 2018-07-13

**Authors:** Even S. Riiser, Thomas H. A. Haverkamp, Ørnulf Borgan, Kjetill S. Jakobsen, Sissel Jentoft, Bastiaan Star

**Affiliations:** ^1^Centre for Ecological and Evolutionary Synthesis, Department of Biosciences, University of Oslo, Oslo, Norway; ^2^Department of Mathematics, University of Oslo, Oslo, Norway

**Keywords:** Atlantic cod, intestinal microbiome, gut microbiome, microbiota, 16S rRNA, *Vibrionales*, *Photobacterium*

## Abstract

Atlantic cod (*Gadus morhua*) provides an interesting species for the study of host-microbe interactions because it lacks the MHC II complex that is involved in the presentation of extracellular pathogens. Nonetheless, little is known about the diversity of its microbiome in natural populations. Here, we use high-throughput sequencing of the 16S rRNA V4 region, amplified with the primer design of the Earth Microbiome Project (EMP), to investigate the microbial composition in gut content and mucosa of 22 adult individuals from two coastal populations in Norway, located 470 km apart. We identify a core microbiome of 23 OTUs (97% sequence similarity) in all individuals that comprises 93% of the total number of reads. The most abundant orders are classified as *Vibrionales, Fusobacteriales, Clostridiales*, and *Bacteroidales*. While mucosal samples show significantly lower diversity than gut content samples, no differences in OTU community composition are observed between the two geographically separated populations. All specimens share a limited number of abundant OTUs. Moreover, the most abundant OTU consists of a single oligotype (order *Vibrionales*, genus *Photobacterium*) that represents nearly 50% of the reads in both locations. Our results suggest that these microbiomes comprise a limited number of species or that the EMP V4 primers do not yield sufficient resolution to confidently separate these communities. Our study contributes to a growing body of literature that shows limited spatial differentiation of the intestinal microbiomes in marine fish based on 16S rRNA sequencing, highlighting the need for multi-gene approaches to provide more insight into the diversity of these communities.

## Introduction

Complex and specialized gut bacterial communities, collectively called the intestinal microbiome, provide a multitude of biological functions in fish [reviewed in (Izvekova et al., 2007; Nayak, 2010; Sullam et al., 2012; Ghanbari et al., 2015; Wang et al., 2017)]. For instance, the intestinal microbiome is essential in processes like fermentation in the hindgut of herbivorous species (Clements et al., 2009), innate immunity (Rawls et al., 2004; Goméz and Balcázar, 2008), vitamin synthesis (Sugita et al., 1991) and influences expression of numerous host genes (Falcinelli et al., 2015). Despite these important biological roles, the diversity of the intestinal microbiome in natural populations remains unknown for many fish species. Studying microbial diversity in wild populations of teleosts provides valuable information regarding the factors affecting establishment and final composition of the fish gut microbiome (Lescak and Milligan-Myhre, 2017). This likely involves a complex interaction between exogenous factors (diet, salinity, etc.) and endogenous factors (genotype, trophic level, etc.) (Nayak, 2010; Romero et al., 2014). Comparative analyses of different fish species indicate that environmental variables (i.e., in salinity and trophic level) and fish taxonomy in particular contribute to the community composition in fish (Sullam et al., 2012). Intriguingly, in Atlantic salmon (*Salmo salar*) the bacterial community composition is not significantly different in populations sampled from both sides of the Atlantic ocean (Llewellyn et al., 2016). Whether such low spatial differentiation is the norm in other marine fish remains largely unexplored.

Atlantic cod (*Gadus morhua*) is an ecologically and commercially important species of the North Atlantic Ocean and represents a unique study system of the fish gut microbiome for several reasons. First, the gadiform adaptive immune system lacks the Major Histocompatibility Complex (MHC) II, which may affect its host-microbe interactions (Star et al., 2011; Star and Jentoft, 2012; Malmstrøm et al., 2016). Second, this omnivore is exposed to a variety of environmental conditions due to its ability to exploit a wide range of ecological niches (Righton et al., 2010). It has a large spatial distribution, which comprises various subpopulations with divergent migratory behavior (Cohen et al., 1992). Third, the intestinal wall of Atlantic cod contains a large number of goblet cells producing prolific levels of mucosa, compared to Atlantic salmon (Inami et al., 2009). The dense mucosal layer provides an opportunity for comparative analyses of microbial communities in the intestinal content vs. those of the mucosal layer (Ringø et al., 2016). Finally, there is an increasing interest in the aquaculture of Atlantic cod (Johansen et al., 2009), and an improved understanding of its natural microbial diversity can provide important baseline data (Lescak and Milligan-Myhre, 2017).

Culture-based studies have demonstrated that the microbiome of Atlantic cod is dominated by *Vibrionales*, *Bacteroidales*, *Erysipelotrichales* and *Clostridiales* (Ringø et al., 2006; Dhanasiri et al., 2011). Nevertheless, high inter-individual variation in the intestinal bacterial community composition has been observed among reared cod-larvae (Fjellheim et al., 2012). Similarly, inter-individual variation has been observed at a single location, using culture-independent 16S rRNA sequencing (Star et al., 2013). These observations suggest that the final community composition is the result of a stochastic process during bacterial colonization, although little is known about the natural variation of the intestinal bacterial communities over larger geographical scales in Atlantic cod.

**FIGURE 1:**
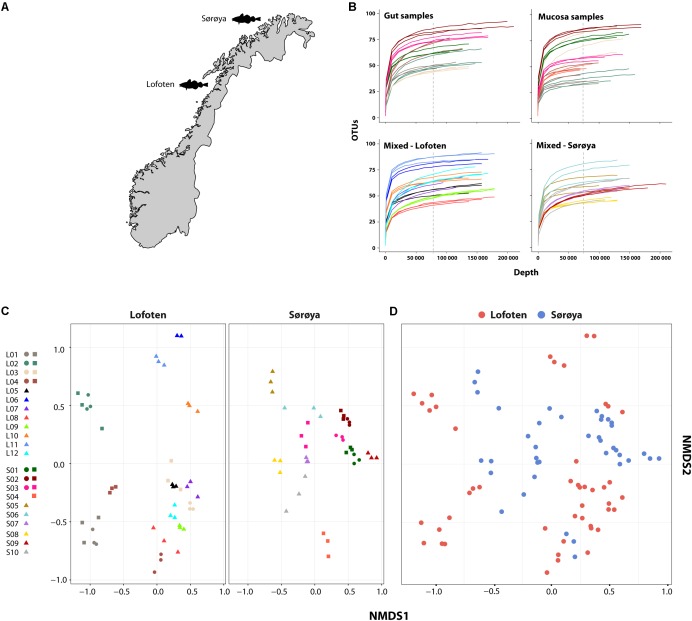
Microbial intestinal communities of wild Atlantic cod. **(A)** Map of sampling locations **(B)** Rarefaction curve analysis showing the number of detected OTUs as a function of read number (depth) for 22 Atlantic cod specimens. For seven specimens (upper two curve plots) gut and mucosal samples were successfully collected from the same individual and sequenced. Sequences are clustered using a 97% similarity cut-off. Replicates (3 per individual, except 2 for L06) are colored identically within each plot. Gut and mucosal samples collected from the same individual are colored identically. Color corresponds to the legend in **(C)**. All curves are based on non-normalized sequence read counts. Low-abundant OTUs are filtered based on a 0.001% minimum abundance threshold (see “Methods”). The vertical dotted line indicates the depth to which the read counts were normalized (74,374 reads). **(C)** Non-metric multidimensional scaling (NMDS) plot of all samples based on Bray-Curtis dissimilarity. Samples are plotted separately per location. Replicates from the same individual are colored identically. L = Lofoten, S = Sørøya. Gut (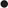), mucosa (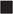), and mixed (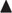). **(D)** Non-metric multidimensional scaling (NMDS) plot of Lofoten (red) and Sørøya (blue) based on Bray-Curtis dissimilarity. The stress value of the NMDS plots in **(C)** and **(D)** is 0.226.

Our goal is to investigate the intestinal bacterial community composition in two coastal Atlantic cod populations located 470 kilometers apart using culture-independent high-throughput sequencing of the 16S rRNA V4 region. We compare the taxonomic composition and diversity of the Atlantic cod intestinal microbiome between two locations (Lofoten and Sørøya, **Figure 1A**). Additionally, we explore the potential to separate “resident” vs. “transient” microbiota by dividing a number of individual samples by tissue type, i.e., gut content versus mucosal layer. We find that a single 16S rRNA *Vibrionales* oligotype (genus *Photobacterium*) comprises nearly 50% of the reads in both locations, and show that the intestinal bacterial community is numerically dominated by a limited amount of highly abundant 16S rRNA oligotypes. Although we detect a significantly lower diversity in mucosal samples, we observe no significant differences in community composition between the two locations.

## Materials and Methods

### Sample Collection

Wild coastal Atlantic cod specimens were collected in Lofoten (N68.0619167, W13.5921667) (12 individuals, August 2014) and Sørøya (N70.760418, W21.782716) (10 individuals, September 2013). A 3 cm long part of the hindgut (immediately above the short, wider rectal chamber) was aseptically removed *post-mortem* by scalpel and stored on 70% ethanol. The samples were frozen (-20°C) for long-term storage. Otoliths were collected for age determination, and relevant metadata such as length, weight, sex, and maturity were registered (Supplementary Table S1).

### Sample Handling and DNA Extraction

Eight individuals (4 from Lofoten, 4 from Sørøya) were randomly selected for analysis of the intestinal content vs. mucosal layer. These intestinal samples were split open lengthwise, before the gut content was gently removed using a sterile disposable spatula, after which the mucosal layer was scraped off the gut lining with another spatula. For each of the remaining 14 individuals (8 from Lofoten, 6 from Sørøya), the gut content and mucosal layer were combined. For each extraction source (gut content, mucosa or mixture) per individual, three technical replicates were obtained for downstream processing (independent DNA extraction and PCR barcoding). Briefly, each individual sample was washed in 500 μl 100% EtOH and centrifuged before the ethanol was allowed to evaporate, after which dry weight was measured before proceeding to DNA extraction. Finally, the 22 individuals yielded eight gut content samples (in triplicate, G1-G3), eight mucosal samples (in triplicate, M1-M3) and 14 mixed samples (in triplicate, Mi1-Mi3). We also included one positive control consisting of DNA extract from an *Escherichia-Shigella* colony and two negative controls (extraction blanks) for a total of 93 samples. DNA was extracted from between 10 and 640 mg dry weight of gut content or mucosa using the *MoBio Powersoil HTP 96 Soil DNA Isolation Kit* (MoBio Laboratories, Carlsbad, CA, United States) according to the DNA extraction protocol (v. 4.13) utilized by the Earth Microbiome Project (EMP) (Gilbert et al., 2010). DNA was eluted in 100 μl Elution buffer and stored at -20°C.

### Sequence Data Generation

The V4 region of the 16S rDNA gene was amplified according to a slightly modified version of the EMP protocol (original v. 4.13) (Gilbert et al., 2010) using the primers 515f (5^′^-GTGCCAGCMGCCGCGGTAA-3^′^) and 806r (5^′^-GGACTACHVGGGTWTCTAAT-3^′^), the latter containing a 12 bp barcode unique for each sample (Supplementary Table S2). Reactions were amplified for 30 cycles (3 min at 94°C, 30 cycles of 45 s at 94°C, 60 s at 52°C, and 90 s at 72°C), with a primer extension of 10 min at 72°C. In contrast to the original EMP protocol, each extraction template was amplified *once*, including the negative extraction controls. PCR amplification was verified on a 1% agarose gel. The PCR products were normalized using the *SequalPrep Normalization Plate Kit* (Invitrogen, Paisley, United Kingdom) eluted in 20 μl Elution Buffer and the 96 Illumina compatible libraries were pooled. The pool was concentrated and purified using the *Amicon Ultra-0.5 mL Centrifugal Filter 30K Device* system (Merck KGaA, Darmstadt, Germany). Finally, the amplicons were paired-end sequenced by StarSEQ (StarSEQ GmbH, Mainz, DE) using Illumina MiSeq (Illumina, San Diego, CA, United States) V3 chemistry with the addition of 20% PhiX, allowing a maximum of 1 bp mismatch during demultiplexing. All sequence data have been deposited in the European Nucleotide Archive (ENA) under study accession number PRJEB22384 (Riiser, 2017).

### 16S rRNA Amplicon Processing

Read qualities were assessed using *FastQC* (ver. 0.11.2) (Andrews, 2010) and primer and adapter sequences were removed using *cutadapt* (ver. 1.8.1) (Martin, 2011) (–cut; hard cutoff of 48 first base pairs). Remaining phiX sequences were removed by mapping reads to the phiX reference genome [GenBank:J02482.1] using *BWA* (ver. 0.7.13) (Li and Durbin, 2009) and discarding matching sequences using *seqtk* (ver. 2012.11) (Li, 2012). Downstream processing and sequence analysis was performed using *Mothur* (ver. 1.36.1) (Schloss et al., 2009) according to the Mothur Illumina MiSeq SOP. Briefly, paired-end reads were joined using the command *make.contigs* and filtered based upon a minimum average Phred quality score of 23, a maximum contig length of 375 bp and a zero-tolerance to ambiguous base pairs. Chimeras were removed using the *UCHIME* algorithm (Edgar et al., 2011) implemented in *Mothur*, before the sequences (V4 region) were aligned and classified using the Silva SEED bacterial database (ver. 119) as reference (Pruesse et al., 2007). The dataset was clustered into OTUs based on a 97% sequence similarity using average neighbor clustering. The OTU table output of *Mothur* was imported into *Rstudio* (ver. 0.99.893) (Racine, 2010) [based on R (ver. 3.2.3) (R Core Team, 2017)] for further processing using the R package *phyloseq* (ver. 1.14.0) (McMurdie and Holmes, 2013). Results were visualized using the R package *ggplot2* (ver 2.0.0) (Wickham, 2009).

OTU counts were normalized using a common scaling procedure, following McMurdie and Holmes (2014). This involves multiplying every OTU count in a given library with a factor corresponding to the ratio of the smallest library size in the dataset to the library size of the sample in question, replacing rarefying (i.e., random sub-sampling to the lowest number of reads). Normalizing using this procedure effectively results in the library scaling by averaging an infinite number of repeated sub-samplings. Filtering was performed to reduce spurious OTUs caused by PCR- or sequencing errors. A minimum abundance threshold of 0.001% of the total number of reads in the dataset was defined, corresponding to 64 reads. Any OTU not exceeding this threshold in at least one sample was removed. Rarefaction curves, alpha diversity measures and ordination distances for non-metric multidimensional scaling (NMDS) plots were calculated in *phyloseq*. Distances were calculated based on OTUs with more than 10 reads (> 99% of total number of reads).

### Oligotyping

Investigation of any concealed diversity within OTUs was performed by oligotyping (ver. 2.0) (Eren et al., 2013). This is a supervised computational method to distinguish closely related but distinct bacterial organisms. In short, an oligotype is a concatenation of the most information-rich single-nucleotide polymorphisms (SNPs) in a genetic marker sequence. A Shannon entropy analysis of the aligned 16S rRNA V4 sequences identifies information-rich positions, and a user-selected set of these are chosen for further analysis by visual inspection according to the Eren et al., 2013 paper. Oligotype profiles are then generated through a process involving different filtering parameters. Due to the supervised nature of the method, we limited the oligotyping to the ten most highly abundant OTUs in our dataset. The following parameters were applied: *s* = 4, *a* = 0.1, *A* = 0, and *M* = 176 (mean library size/1000).

### Statistical Analysis

Differences in within-individual diversity (alpha diversity) were studied using a linear mixed effects model with random effect for fish, following Zuur et al., 2009, chapter 5.3. The “optimal” models (i.e., the models that best describe the individual diversities) were identified by a top-down reduction strategy and selected through *t*-tests based on Restricted Maximum Likelihood (REML) estimation. Model assumptions were verified through plotting of residuals. The effect size of the variables on the diversity measures was determined by comparing the estimate of that variable to the interquartile range (middle 50% of the spread) of the diversity measure. For the sake of simplicity, we defined the statistical variable “tissue” to contain both mucosal tissue and gut content, although the latter in not a tissue *per se*. Differences in bacterial community composition (beta diversity) between gut content and mucosal tissue, and between the two locations, were assessed using Permutational Multivariate Analysis of Variance (PERMANOVA) using the *adonis* function in the R package *vegan* (ver. 2.4-1) (Oksanen et al., 2017). The test was run with 20,000 permutations, applying both OTU-based (Bray-Curtis/Jaccard) and phylogeny-based (weighted and unweighted UniFrac) distance measures. For location analysis, the mean normalized abundance for each of the 139 OTUs in the 22 individuals was applied. For tissue analysis, normalized abundance of all gut and mucosa replicates (*n* = 42) was used. Due to dependency within replicates for each fish, individuals were used as strata (blocks) in the gut vs. mucosa testing. PERMANOVA assumes the multivariate dispersion in the compared groups to be homogeneous; this was verified using the *betadisper* function (*vegan*). Similarity percentage (SIMPER) procedure implemented in *vegan* was used to quantify the contribution of individual OTUs to the overall Bray-Curtis dissimilarity between gut content and mucosa. OTUs with a statistically significant differential abundance between the locations were determined using *MetaStats* (White et al., 2009). Only OTUs containing more than 0.1% of the total number of reads in the normalized and filtered dataset (of 139 OTUs) were used in this analysis. Finally, differences in relative abundance of the individual oligotypes between Lofoten and Sørøya were studied using linear mixed effects models for log-transformed read counts, and selected and verified as described above, with individuals and oligotypes (within individuals) treated as random effects.

## Results

We obtained 14,944,840 paired-end reads that were assembled based on sequence overlap. Of the original 93 samples, this dataset contained 86 cod and three control libraries (four replicates failed to sequence). The libraries (excl. controls) had a median size of 173,377 reads per replicate (Supplementary Table S3). The positive control contained 96.5% sequences classified as *Escherichia-Shigella*, as expected. The negative controls yielded less than 0.01% of total number of reads in the dataset (Supplementary Table S4). These reads represent known reagent and laboratory contaminant OTUs (Salter et al., 2014). Due to insufficient sequencing of a Sørøya sample (S04, all three replicates), only seven individuals (4 Lofoten, 3 Sørøya) were used for comparison of diversity in gut and mucosa.

After normalizing OTU abundances to the size of the smallest library (74,374 reads) by common scaling, and filtering of OTUs based on abundance, 139 OTUs representing 98.6% of the dataset were identified (Supplementary Table S5). Rarefaction curve analyses on the normalized data show that variation in the number of OTUs detected per sample is not caused by uneven sequencing depth (**Figure 1B**). The technical replicates cluster close to one another per respective tissue type and individual specimen in a multivariate NMDS analyses (**Figure 1C**), and this methodological consistency is reflected by the similar OTU composition of the individual replicates (Supplementary Figure S1). Finally, we observe a large overlap between the OTU community profiles when clustering individuals from both locations together (**Figure 1D**).

### Taxonomic Composition of the Bacterial Gut Community

**FIGURE 2:**
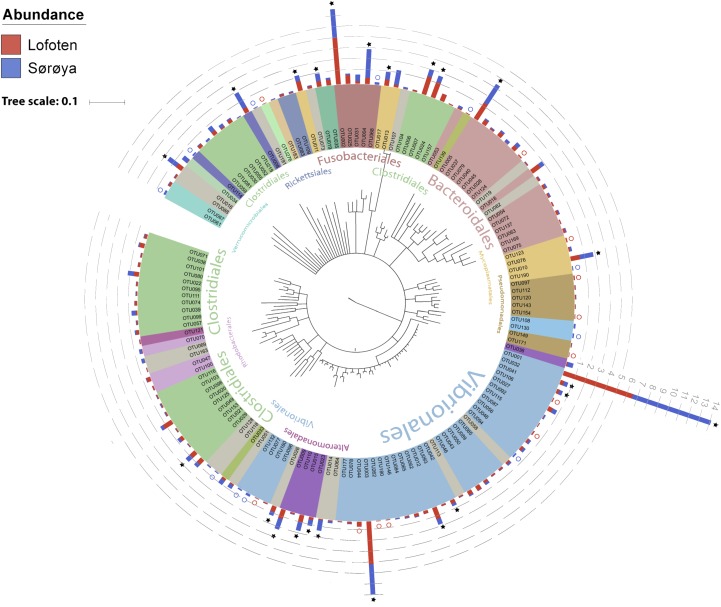
Phylogenetic tree of the Atlantic cod intestinal bacterial community members and their relative abundances at two sample locations. Phylogenetic tree of the 16S rRNA V4 sequences from the 139 OTUs in the Atlantic cod intestines. The Neighbor-joining tree was generated based on the Jukes-Cantor model of nucleotide-substitution, using *Geneious* (ver. 10.2.2). OTUs are colored based on an order-level classification, with unclassified OTUs in gray. Stacked bars represent relative read abundance (square root transformed, gray dashed lines) of each OTU in Lofoten (red) and Sørøya (blue). Some OTUs are found in all specimens (stars), whereas others (colored circles) are observed in only one of the locations. Classifications of the largest orders (highest numbers of OTUs) are given in the inner part of the circle. A detailed taxonomic classification is given in Supplementary Table S5.

**FIGURE 3:**
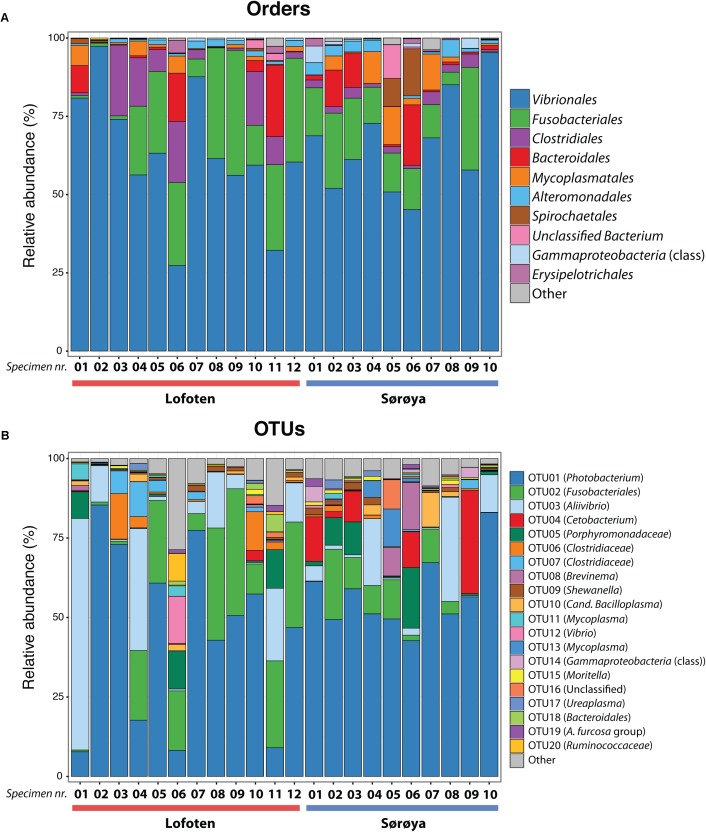
Taxonomic composition of the intestinal microbiome in Atlantic cod specimens from Lofoten and Sørøya. **(A)** Relative abundance of reads classified to bacterial orders. Colors represent the 10 most abundant orders. Numbers 1–12 and 1–10 represent individual specimens. **(B)** Relative abundance of reads by OTUs (at 97% similarity) classified to lowest identifiable taxonomic level. Remaining orders or OTUs are merged into “Other” categories (gray bars). The relative abundances of replicate samples or tissues were averaged per individual. The taxonomic composition of each individual replicate is depicted in Supplementary Figure S1.

The 139 OTUs are binned into 9 phyla, 14 classes and 20 orders (**Figure 2** and Supplementary Table S6), with all the abundant OTUs having been identified at both locations. 23 OTUs are detected in one of the locations only (**Figure 2**), of which 17 belong to the 30% least abundant OTUs (Supplementary Table S5). A core microbiome of 23 OTUs is identified based on shared membership in all 22 individuals (**Figure 2** and Supplementary Table S7). This core community includes the ten most abundant OTUs in the dataset, and represents about 93% of the total number of reads. The *Proteobacteria* represents 67% of the total read count (63 OTUs), followed by *Fusobacteria* (15.7%/5 OTUs), *Firmicutes* (6.1%/33 OTUs) and *Bacteroidetes* (4.5%/18 OTUs). *Gammaproteobacteria* represents the major bacterial class, with a median relative abundance of 64.6% among the samples. This is mainly due to the *Vibrionales* order; with a relative abundance ranging from 27 to 97% it is clearly the most prolific member of the intestinal microbiome in northern coastal Atlantic cod (**Figure 3A**). *Fusobacteriales*, *Clostridiales* and *Bacteroidales* are abundant in some, but not all individuals.

### Dominant Members of the Gut Microbiome

The ten most abundant OTUs in the Atlantic cod individuals represent 88.6% of the total number of sequence reads (**Table 1**). The most abundant OTU (OTU 01), a *Photobacterium*, represents 78% of all *Vibrionales* reads, and comprises 50% of all sequences in the study. It also constitutes > 50% of the reads in 13 of the 22 specimens (**Figure 3B**). In five of the seven specimens used for analysis of differences between gut content and mucosa, OTU 01 represents > 45% of the reads (mean of triplicates) in both gut content and mucosa (Supplementary Figure S2). Blast results provide no clear species-level identification of this OTU due to multiple entries of different species. In the cases that OTU 01 is not the most prolific member, other common OTUs (such as OTU 03 (*Aliivibrio*), or OTU 02 (*Fusobacteriales*)) show a higher abundance (**Figure 3B**).

**Table 1:** The 10 most abundant OTUs in the Atlantic cod samples.

OTU nr.	Taxonomy	Total reads	Relative abundance (%)
01	*Photobacterium*	2 436 281	50.36
02	*Fusobacteriales*	635 730	13.14
03	*Aliivibrio*	576 357	11.91
05	*Porphyromonadaceae*	161 547	3.46
04	*Cetobacterium*	167 536	3.34
06	*Clostridiaceae*	85 810	1.77
07	*Clostridiaceae*	65 527	1.35
08	*Brevinema*	59 540	1.23
10	*Cand. Bacilloplasma*	43 301	1.12
09	*Shewanella*	54 358	0.89

*The table shows the OTU name, the lowest possible classified taxonomic level, the number of reads after normalization and the relative abundance of the OTU in the cod community*.

### Intestinal Microbial Diversity

**FIGURE 4:**
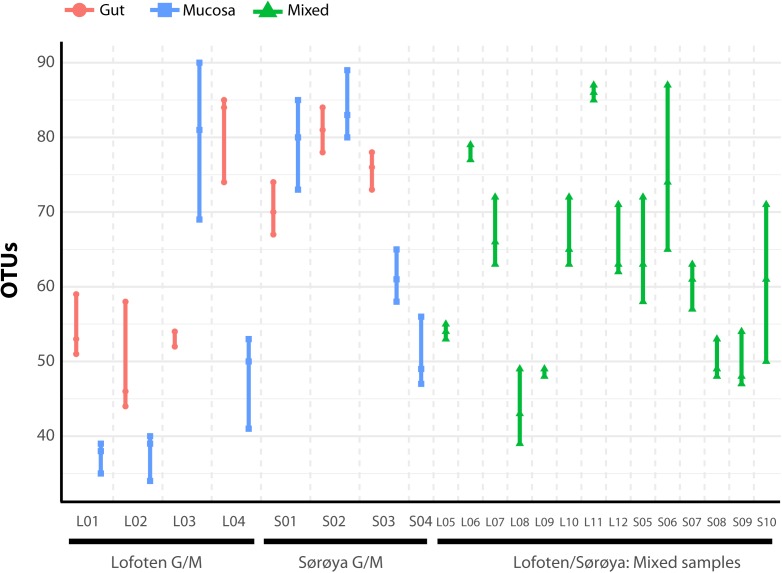
Number of observed OTUs in 22 Atlantic cod samples. Each of the 86 replicates is represented by a point. Individuals sampled for both gut (red) and mucosa (blue) on the left half of the figure, individuals sampled for a mix (green) of gut and mucosa on the right half.

The individual tissue samples contain between 34 and 90 OTUs (**Figure 4**), and vary also in diversity estimated by Shannon (H) and Inverse Simpson (1/D) indexes (Supplementary Figure S3 and Supplementary Table S8). The optimal linear mixed effects models for the three diversity measures, including fish specimen as a random effect, reveal a statistically significant difference between mucosa and gut content (**Table 2** and Supplementary Table S9). For all three measures, the diversity is significantly lower in mucosa than in the gut. However, the estimated differences are small compared to the variation in the diversity measures between all 22 individuals. For the number of OTUs, the estimate in **Table 2** corresponds to 23% of the interquartile range for the variation in OTUs between all fishes, while for the Shannon and Inverse Simpson indexes the estimates correspond to 26 and 42%, respectively, of the interquartile range. The reason why such small differences become significant, is that we have triplicates of both gut and mucosa samples for seven individuals (Supplementary Figure S2), ensuring high statistical power for the comparison of gut vs. mucosa. For the individuals with mixed tissue triplicates, we do not have any other types of tissue samples. Therefore, we have much lower statistical power for comparing mixed tissue with the other tissue types. As a result, we do not find a significant effect of mixed tissue even though the estimates for two of the diversity measures are of the same order of magnitude as for mucosa. The results in **Table 2** also show that length and sex of the Atlantic cod specimens have a significant impact on the observed number of OTUs. There are no significant differences in within-sample diversity between the two locations (Supplementary Table S9).

**Table 2:** Effects of covariates on intestinal diversity (alpha diversity).

	OTUs		Shannon		Inverse simpson	
	Estimate	*p*-value	Estimate	*p*-value	Estimate	*p*-value
Intercept	80.38	0.000	1.44	0.000	2.88	0.000
Tissue mixed	-6.50	0.220	0.06	0.789	0.50	0.572
Tissue mucosa	-5.62	0.046*	-0.15	0.000*	-0.53	0.000*
Length (cm)	-0.35	0.032*				
Sex male	11.40	0.028*				

*Results from the optimal linear mixed effects models used to analyze within-sample (alpha) diversity. “Estimate” indicates the estimates of the regression coefficients for the fixed effects in the model. Tissue estimates are given relative to gut, and the sex estimate is given relative to females. Significant effects (*p* < 0.05) of covariates are indicated by an asterisk*.

Results from between-sample (beta) diversity analyses agree with those from the within-sample (alpha) analyses. PERMANOVA analysis reveals a statistically significant difference in community composition between gut and mucosa using Bray-Curtis, Jaccard and Weighted UniFrac diversity measures, while there is no significant difference between Lofoten and Sørøya (**Table 3**). Nevertheless, the difference between gut content and mucosa has a very small effect size, and can only explain about 1–3% of the total variation in the gut and mucosal samples. The reason why these small effects become significant is, as described above, that we have high statistical power for comparison of gut vs. mucosa. The significant difference between gut content and mucosa is preserved when limiting the analysis to the five most abundant OTUs (Supplementary Table S10), indicating that a restricted number of highly abundant OTUs is responsible for the differences. This is in agreement with results from the SIMPER analysis, where the five most abundant OTUs (OTU 01–05) together contribute 77.3% to the observed (Bray-Curtis) dissimilarity between gut and mucosa (Supplementary Table S11). MetaStats analysis of differential abundance between the locations identifies four OTUs -*Fusobacteriales* (OTU 02), *Cetobacterium* (OTU 04), *Gammaproteobacteria* (OTU 14) and *Shewanella* (OTU 23) –with a low *p*-value (**Table 4**), although no significance remains after Bonferroni correction.

**Table 3:** PERMANOVA analysis of diversity differences between bacterial communities (beta diversity).

		Bray–Curtis	Jaccard	Unweighted uniFrac	Weighted uniFrac
Location	R2	0.11	0.09	0.07	0.07
	*p*-value	0.047	0.041	0.146	0.218
Tissue	R2	0.01	0.01	0.03	0.01
	*p*-value	0.001*	0.001*	0.015	0.003*


*Location: 21 degrees of freedom (df), tissue: 41 df. Significant effects (Bonferroni corrected, *p* < 0.00625) are indicated by an asterisk*.

**Table 4:** OTUs differentially abundant between Lofoten and Sørøya.

	Lofoten		Sørøya				
OTU nr.	Mean	SE	Mean	SE	*p*-value	Total reads^∗^	Taxonomy
**02**	**18.78**	4.24	7.13	2.34	0.030	635 730	*Fusobacteriales*
**04**	0.32	0.28	**7.06**	3.36	0.034	161 547	*Cetobacterium*
**14**	0.01	0.00	**1.00**	0.55	0.005	21 946	*Gammaproteobacteria*
**23**	0.06	0.03	**0.32**	0.12	0.045	8 322	*Shewanella*


*41 OTUs, each representing more than 0.1% of the total abundance, were analyzed using *MetaStats*. Mean and SE values are in percent. None of the OTUs give *p*-values below the Bonferroni corrected *p*-value (0.05/41 = 0,001) significance threshold. Bold values mark the location of highest mean abundance. ^∗^Total abundance of OTU in all samples*.

### Identification of Oligotypes With Differential Abundance

**FIGURE 5:**
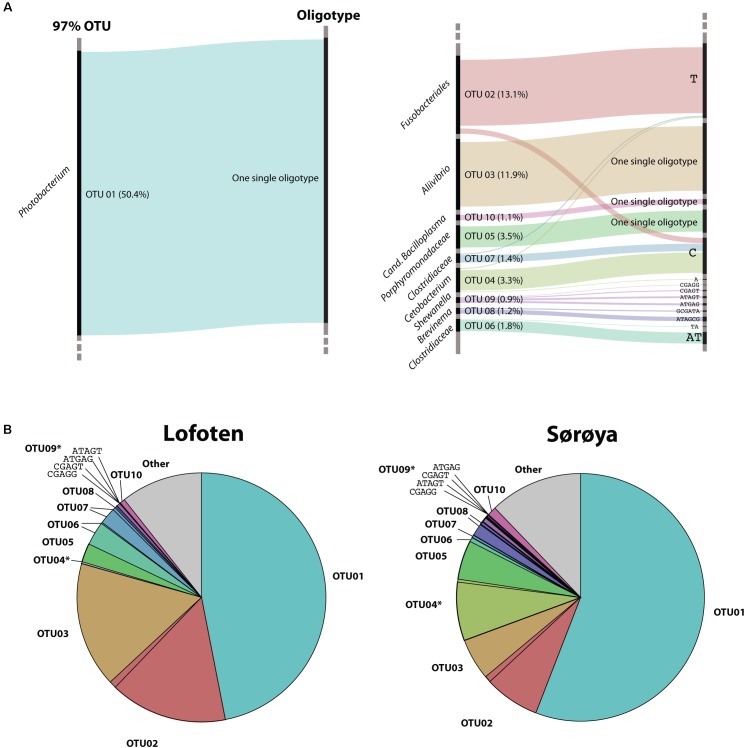
Oligotypes among the most abundant OTUs. **(A)** Substructure of the ten most abundant OTUs in the gut microbiome of Atlantic cod. Black bars on left side represents OTUs, while bars on the right side represents unique oligotypes. The height of a colored bar represents its overall abundance, also stated in the parenthesis behind each OTU. Genus (or lowest classified taxonomic level) is given to the left of each OTU. For four OTUs, no additional oligotypes are detected, and the most abundant OTU (OTU01, left) consists of a single oligotype. **(B)** Relative abundance of OTUs and their representative oligotype(s) in the gut microbiome of Atlantic cod in two locations. Colors represent different OTUs, and identically colored sectors represent different oligotypes within an OTU. OTUs containing differentially abundant oligotypes as determined by mixed effects modeling are marked with an asterisk (^∗^). For illustrational purposes, the four oligotypes of OTU09 are labeled in the figures.

Oligotyping reveals higher taxonomical detail in six of the ten most abundant OTUs (**Figure 5A**). Each of the OTUs 02, 04 and 06–09 consist of at least two oligotypes, while the remaining four OTUs show no such substructures (i.e., there is one oligotype per OTU). Of the six OTUs with multiple oligotypes, OTU 04 and OTU 09 contain oligotypes with a differential abundance between the two locations. OTU 04 oligotype “C” has a small but significantly higher abundance in Sørøya than in Lofoten (**Table 5**). *P*-values just above 5% of an additional OTU 04 oligotype (“T”) and two OTU 09 oligotypes (“ATGAG/CGAGT”) weakly indicate a differential abundance between the two locations. The remaining OTUs with multiple oligotypes contain no differentially abundant oligotypes (**Figure 5B**).

**Table 5:** Differential abundance of individual oligotypes.

OTU nr.	Genus	Oligotype	Estimate	*p*
04	*Cetobacterium*	C	3.37	0.003^∗^
		T	1.70	0.051.
		A	-0.84	0.316
09	*Shewanella*	ATGAG	1.77	0.055.
		ATAGT	-1.00	0.309
		CGAGT	1.70	0.057.
		CGAGG	0.44	0.603


*“Estimate” indicates the estimated effect of Sørøya compared Lofoten. The estimates are obtained from a linear mixed effects models for log-transformed read counts (with one added to all read counts) with individuals and oligotypes (within individuals) as random effects, and tissue type, oligotype, location and interaction between oligotype and location as fixed effects. OTU04 oligotype C has a small but significantly higher abundance in Sørøya than in Lofoten (*p* < 0.05, ^∗^). P-values just above 5% of three other oligotypes (.) weakly indicate a differential abundance between Lofoten and Sørøya*.

## Discussion

Here, we have investigated the diversity and taxonomic composition of the gut microbiome of Atlantic cod from two separate locations along the Norwegian coastline based on 16S rRNA sequencing. The Atlantic cod gut community is dominated by *Proteobacteria*, *Fusobacteria*, *Firmicutes* and *Bacteroidetes* (Supplementary Table S6)*;* all known to be among the most dominant phyla in the gastrointestinal tract of marine fish (Romero et al., 2014; Wang et al., 2017). *Vibrionales*, *Fusobacteriales*, *Clostridiales* and *Bacteroidales* are among the most abundant orders. In particular, the order *Vibrionales* occurs in high abundance (64% of reads), which is in agreement with what was found in a meta-analysis of marine fish microbiomes (Sullam et al., 2012), in a laboratory experiment in which Atlantic cod were exposed to varying levels of oil (Bagi et al., 2018), and in the intestinal microbiome of 11 wild Atlantic cod individuals caught in a single location in the Oslo Fjord (Star et al., 2013). In the Atlantic cod exposed to oil, *Vibrionales* make up more than 40% of the reads, regardless of treatment, with a single *Photobacterium* OTU representing 71% of the *Vibrionales* sequences (Bagi et al., 2018). While the Oslo Fjord study reported large inter-individual differences in OTU abundance (Star et al., 2013), these differences were largely driven by variation in abundance of the rare members of the microbial community. Yet – similar to what we report here – the Oslo Fjord individuals shared a low number of highly abundant OTUs, with *Vibrionales* representing > 50% of the total number of reads (Star et al., 2013). Unlike previous findings, however, we identify a single *Vibrionales* 16S rRNA oligotype (genus *Photobacterium*, OTU 01) that comprises 50% of the sequencing data. To our knowledge, the occurrence of a single, prolific oligotype on such spatial scale has not been reported before in marine fish.

There are two alternative explanations why we observe no genetic diversity within this abundant oligotype: It is well known that amplicon data provide a limited representation of the underlying biological diversity (Liu et al., 2008; Youssef et al., 2009; Vasileiadis et al., 2012; Shakya et al., 2013; Birtel et al., 2015; Amore et al., 2016; Zhang et al., 2018). We here used a set of amplicon primers designed by the EMP, targeting the 16S rRNA V4 region (Gilbert et al., 2010). The advantage of using these primers lies in their economic application – including well described bioinformatic methods –, and the potential for the direct comparative analyses of microbial diversity from a wide range of environments and ecosystems (Gilbert et al., 2010). Nonetheless, the disadvantage is the possibility that the 16S rRNA V4 region does not capture enough sequence variation to sufficiently resolve the bacterial community diversity within this particular ecosystem, leading to the observation of a limited number of abundant OTUs and 16S rRNA oligotypes in these data.

Alternatively, the presence of a single, dominant *Vibrionales* oligotype represents the colonization and subsequent numerical increase of a single – or closely related – bacterial species. While at this stage it is not possible to exclude the first explanation, there are several observations that indicate that this ubiquitous oligotype abundance is not due to limited methodological resolution of the V4 region. First, we find another abundant *Vibrionales* OTU (OTU 03) that also has a lack of within OTU diversity (**Figure 5A**). This indicates that the V4 region is capable of distinguishing genetic variation within *Vibrionales*. Second, other studies – targeting different 16S rRNA regions – have also observed the occurrence of a low number of highly abundant taxa (including *Photobacterium*) in the fish intestinal microbiome, although these did not resolve taxonomy down the level of oligotypes. For example, based on the 16S rRNA V1/V2 region, gut microflora of three shark species is dominated by a single *Photobacterium* OTU that represents up to 98% of the total shared sequences (Givens et al., 2015). Similarly, based on the full-length 16S rRNA gene, 93% of the sequence data comprised a single *Photobacterium* OTU in the intestines of two wild cold water adapted notothenioid species of the Antarctic Ocean (Ward et al., 2009). Moreover, four captive adults of Atlantic halibut were shown to contain > 70% of luminous *Photobacterium phosphoreum* isolates through culture-dependent methods (Verner-Jeffreys et al., 2003). Finally, based on the 16S rRNA V4 region, little differentiation was observed in the Atlantic salmon gut microbiome in fish populations sampled from both sides of the Atlantic Ocean (Llewellyn et al., 2016). These studies indicate that limited *Photobacterium* diversity is present in the gut microbiome using a variety of methods and suggest that this genus is particularly capable of colonization and proliferation in marine fish (Morita, 1997; Urbanczyk et al., 2011).

We observe a small, but significant difference between the microbial communities in gut content and mucosal tissue. The alpha diversity estimates reveal a slightly less diverse community in mucosa than in gut content, which is in agreement with previous findings in fish (Kim et al., 2007; Dhanasiri et al., 2011). This observation supports a hypothesis assuming that the intestinal mucosa hosts a subset of specialized bacteria compared to what is present in the more heterogeneous gut content of an omnivore. We find that the numerically dominant OTUs are abundant in both gut and mucosa. From this, we derive that these OTUs are associated with the intestinal mucosa, as part of the residential (autochthonous) microbiota. This permanent community is most likely to interact with the host and may serve functionally important purposes, i.e., protective immunity (Izvekova et al., 2007). Nevertheless, the limited community differences we observe between the tissue types may also reflect our sampling methodology; out of necessity, the samples were stored in ethanol for a prolonged amount of time, and separation of mucosa and gut content would most likely have been more efficient by sampling of fresh tissue.

Based on OTU level classification, we find no significant overall bacterial community differentiation between Atlantic cod from Lofoten and Sørøya. The intestinal microbiome in coastal Atlantic cod from Lofoten and Sørøya may be similar due to several reasons. First, a strong host-microbe interdependence based on mutual benefits between Atlantic cod and specialized, residential bacteria would promote congruent gut communities in both locations. This requires Atlantic cod to actively recruit certain bacterial species; such host selection is suggested in adult zebrafish and Atlantic salmon parr (Roeselers et al., 2011; Dehler et al., 2017). Second, environmental factors such as diet, temperature or water bacterioplankton content common for the two locations may contribute to a similar diversity and community structure of the fish gut microbiomes (reviewed in Llewellyn et al., 2014; Wang et al., 2017). While no OTU level differentiation in beta diversity is detected between these two locations, two OTUs containing oligotypes that show limited spatial differentiation are identified. These oligotypes could reflect the limited acquisition of more “local” oligotypes into the Atlantic cod microbiome. Hence, oligotyping may reveal a level of spatial segregation which OTU clustering at the 97% similarity threshold alone would not have detected (Eren et al., 2013).

The Atlantic cod gut microbiome contains considerable amounts of *Fusobacteriales*, *Cetobacterium*, *Aliivibrio*, *Porphyromonadaceae*, *Clostridiaceae*, *Brevinema*, and *Shewanella*, which have been observed in fish intestines in other studies (Hansen and Olafsen, 1999; Shiina et al., 2005; Larsen et al., 2014; Gajardo et al., 2016). However, *Photobacterium* (OTU 01) is the predominant oligotype in both gut and mucosal samples from both locations. A high abundance of this oligotype in mucosa suggests it is a residential bacterium associated with the gut lining, and thus potentially involved in host-microbial co-evolution. For example, some *Photobacterium* species show antagonistic activity toward bacterial pathogens in Atlantic cod, *Vibrio anguillarum* and *Aeromonas salmonicida* (Caipang et al., 2010) and hence contribute to protective immunity in Atlantic cod. Considering the loss of the MHC II pathway of the adaptive immune system, the Atlantic cod could therefore benefit from housing *Photobacterium* in its intestines.

Atlantic cod is a dietary generalist that consumes a varied diet. Such dietary behavior might intuitively lead to an expectation of an associated diverse intestinal microbiota, given an exposure to more bacterial diversity. Our lack of large-scale population differentiation suggests this is not the case in Atlantic cod. Interestingly, a negative association between diet diversity and microbial diversity has been observed in stickleback and perch (Bolnick et al., 2014b). It was hypothesized that generalists have more nutritionally diverse gut environments that sustain a limited number of competitively dominant bacteria at high abundance. Those individuals exposed to a more varied diet are shown to have a substantial increase of *Gammaproteobacteria*. This bacterial class also occurs in high relative abundance in our Atlantic cod samples (i.e., *Photobacterium*) and hence this observed microbiome may indeed reflect that of a dietary generalist.

Another explanation for the lack of differentiation in the microbiome of the Atlantic cod intestine may be related to its lack of MHC class II, a key pathway in the vertebrate adaptive immune system. MHC II is produced in antigen-presenting cells which phagocytize extracellular particles, including bacteria. It is therefore proposed that this pathway is involved in the recognition and management of a complex bacterial community (Bolnick et al., 2014a). The absence of such a regulatory mechanism may lead to a limit in the number of resident bacterial species that can be maintained in its intestines. Indeed, it has been suggested that a lower microbial diversity observed in invertebrates is due to their lack of an adaptive immune system (Mcfall-Ngai, 2007). Thus, the role of the Atlantic cod immune system in the active maintenance of microbial species requires further investigation.

## Conclusion

In this study, we find that the Atlantic cod intestinal microbiome consists of a limited number of abundant 16S V4 rRNA oligotypes, with limited differentiation between intestinal bacterial communities in Lofoten and Sørøya. A significant yet small difference in the community diversity between gut and mucosa suggests that the abundant members of the microbiome are part of the more permanent inhabitants of the gut, that possibly play a role in host-microbe co-evolution. Finally, a single *Photobacterium* oligotype is particularly abundant in the Atlantic cod gut microbiome in the two locations 470 km apart. Future studies – in particular multi-gene approaches involving metagenomic/-transcriptomic shotgun sequencing – are needed to provide more detailed insights into the diversity and functional capacity of the intestinal microbiome of Atlantic cod.

## Availability Of Data and Materials

The data set generated and analyzed for this study is available in the European Nucleotide Archive (ENA), study accession number PRJEB22384, http://www.ebi.ac.uk/ena/data/view/PRJEB22384 (Riiser, 2017).

## Ethics Statement

In order to limit the effect of our sampling needs on populations and individuals, we obtained samples as a byproduct of conventional business practice, and all specimens were caught by commercial vessels, euthanized by local fishermen and were intended for human consumption. Samples were taken post-mortem and no scientific experiments have been performed on live animals. Sampling in this manner does not fall under any specific legislation in Norway, and no formal ethics approval was required. The sampling follows the guidelines set by the “Norwegian consensus platform for replacement, reduction and refinement of animal experiments” (Norecopa guidelines for animal experiments).

## Author Contributions

SJ, BS, and TH conceived and designed the experiments. KJ provided the initial framework for the study. ER and SJ sampled the specimens. ER performed the laboratory work. ER and TH performed data analysis. ØB, TH, ER, and BS interpreted the results. ER and BS wrote the paper with input of all authors. All authors read and approved the final manuscript.

## Conflict of Interest Statement

The authors declare that the research was conducted in the absence of any commercial or financial relationships that could be construed as a potential conflict of interest. The reviewer PG and handling Editor declared their shared affiliation.
