# Supplementary material for: A Single Vibrionales 16S rRNA Oligotype Dominates the Intestinal Microbiome in Two Geographically Separated Atlantic cod Populations
Source: Front Microbiol. 2018 Jul 13;9:1561. doi: 10.3389/fmicb.2018.01561 (PMC6053498; doi:10.3389/fmicb.2018.01561)
Supplement: Supplementary file 12 [file Presentation_1.pdf]

# *Supplementary material*

# A single *Vibrionales* 16S rRNA oligotype dominates the intestinal microbiome in two geographically separated Atlantic cod populations

**Even Sannes Riiser[1*], Thomas H.A. Haverkamp[1], Ørnulf Borgan[2], Kjetill S. Jakobsen[1], Sissel Jentoft[1] and Bastiaan Star[1*]**

*** Correspondence**
Even Sannes Riiser*: e.s.riiser@ibv.uio.no
Bastiaan Star*: bastiaan.star@ibv.uio.no

## 1 Supplementary tables

**Table S1 (xlsx): Metadata**
Data collected for all cod specimens used in the study.

**Table S2 (xlsx): Primers and barcodes**
Primer sequences and unique barcode for each sample.

**Table S3 (xlsx): Library sizes**
Number of forward and reverse sequences per library, including raw reads, reads after removal of PhiX sequences and reads after the *Mothur* pipeline (used for downstream analysis).

**Table S4 (xlsx): Positive and negative controls**
Taxonomic composition and number of raw reads in the positive and the two extraction (negative) controls.

**Table S5 (xlsx): OTU table and classification**
Left part of table: Key information of each OTU after abundance filtering and common scaling, including total number of reads, overall relative abundance and the number of individuals each OTU occur in (frequency). Color codes are given below the table. Middle part: The final OTU table after abundance filtering, common scaling and merging of six replicates per sample into separate individuals. Right part: Classification of each OTU.

**Table S6 (xlsx): Taxa level information**
Key relative abundance values of the different taxa (at all taxonomic levels) in individuals from Lofoten and Sørøya, and in the overall dataset.

**Table S7 (xlsx): Core community**
Core OTUs found in all individuals from Lofoten and Sørøya. Genus or lowest classified taxonomical level shown in the last column. c_ = class, o_ = order, f_ = family.

**Table S8 (xlsx): Alpha diversity values**

Alpha diversity estimates of the Atlantic cod intestinal microbial samples. All samples were normalized by a common scaling procedure (see Methods). OTUs were clustered based on 97% sequence similarity.

**Table S9 (xlsx): Alpha diversity differences - Random intercept model**
Results from the *beyond optimal* model and the final random intercept model fitted through model selection according to Zuur et al. 2009 chapter 5.7*, used in testing for significant differences in alpha diversities within locations and tissues. Significant values (< 0.05) are marked in bold.

*****Zuur, A. et al., 2009. *Mixed Effects Models and Extensions in Ecology with R,* Springer. Available at: http://link.springer.com/book/10.1007%2F978-0-387-87458-6#section=15123&page=1.

**Table S10 (xlsx): PERMANOVA with the five most abundant OTUs only**
Results from PERMANOVA analyses of bacterial community differences using the five most abundant OTUs only. Bacterial community differences (beta diversity) were tested between the different locations, and between gut content, intestinal mucosa and a mixture of the two. P-values < 0.05 are marked in bold. These are still above the Bonferroni threshold (p = 0.00625).

**Table S11 (xlsx): SIMPER results**
SIMPER analysis results showing the contribution of each OTU to the Bray-Curtis dissimilarity between gut content and mucosa. In agreement with the results shown in Additional file 13, the five most abundant OTUs (OTU01 – 05) contribute 77.3% to the observed dissimilarity (see column ”Cumulative contribution”). Significance information is supplied in the two last columns.

## 2 Supplementary figures

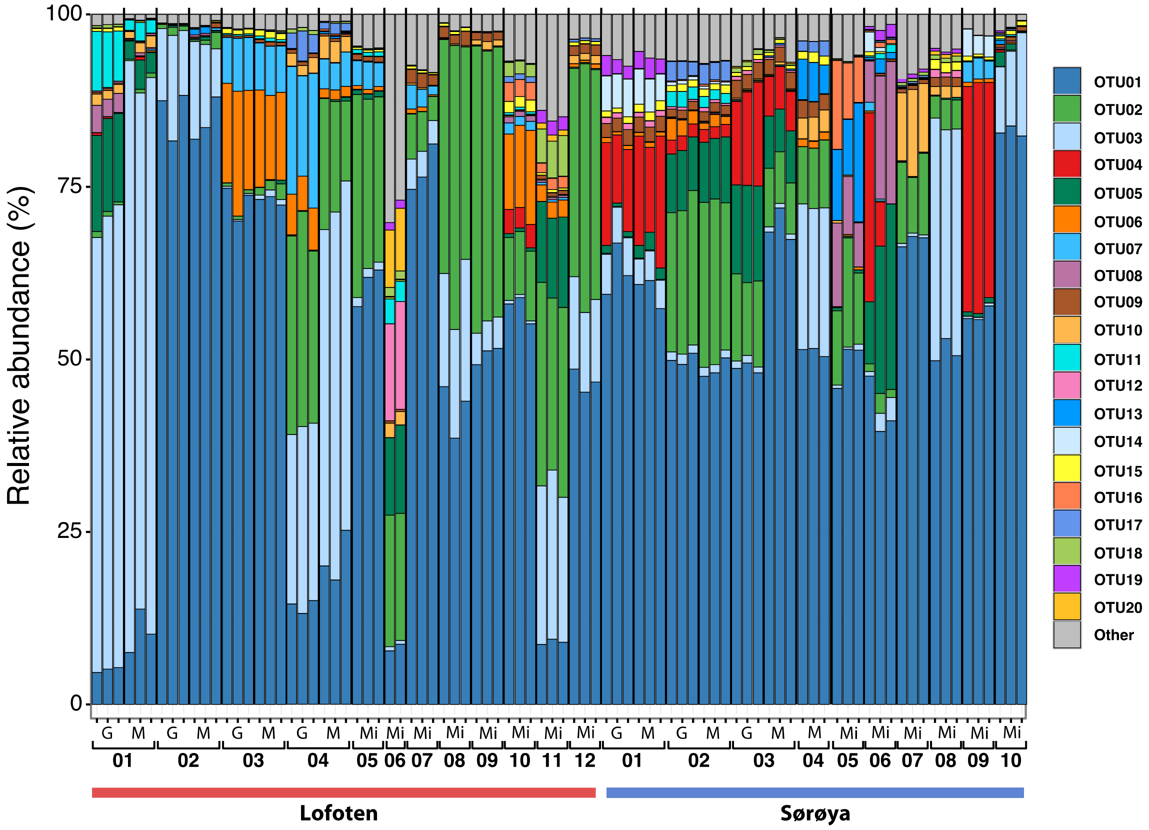


**Figure S1: Relative abundance of OTUs in all 86 replicates.** The 20 most abundant OTUs in cod are colored, while remaining OTUs are merged into the "Other" category (grey bars). G = Gut, M = Mucosa, Mi = Mixed.

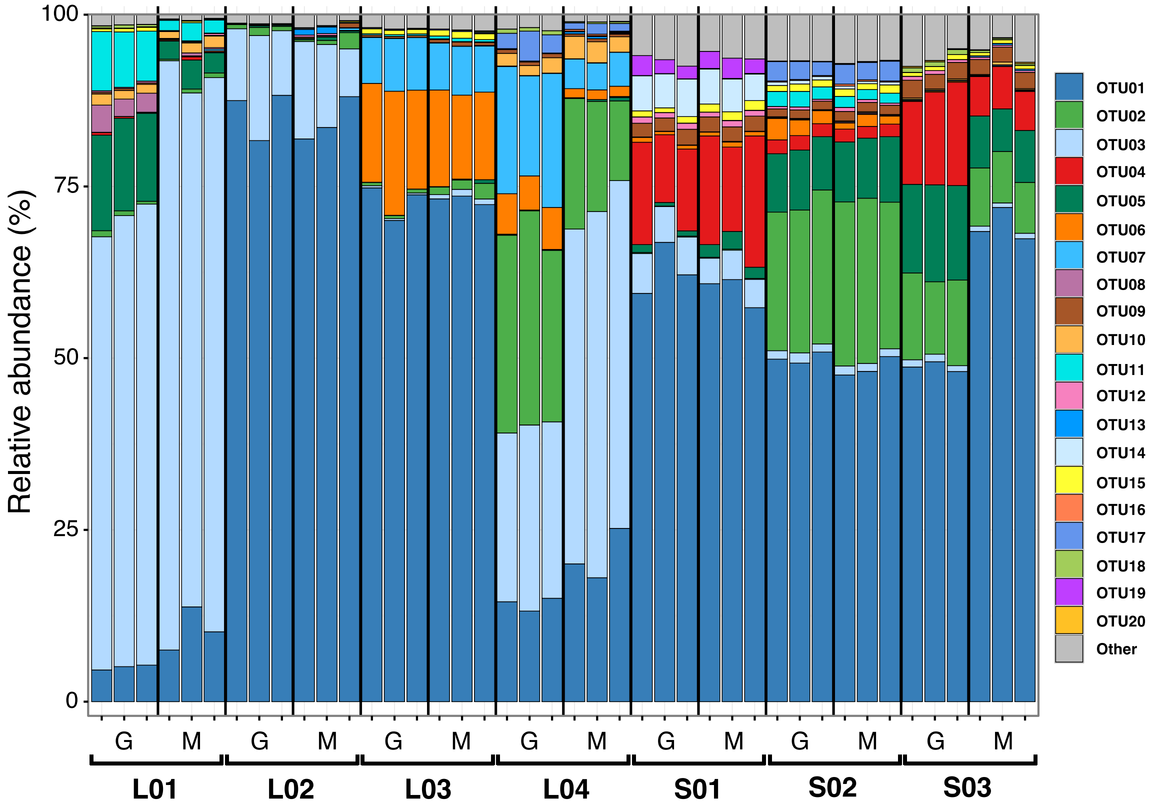


**Figure S2: Relative abundance of OTUs in the gut (G) and mucosal (M) samples.** All replicates from four Lofoten and three Sørøya individuals are shown. The 20 most abundant OTUs in cod are colored, while remaining OTUs are merged into the "Other" category (grey bars).

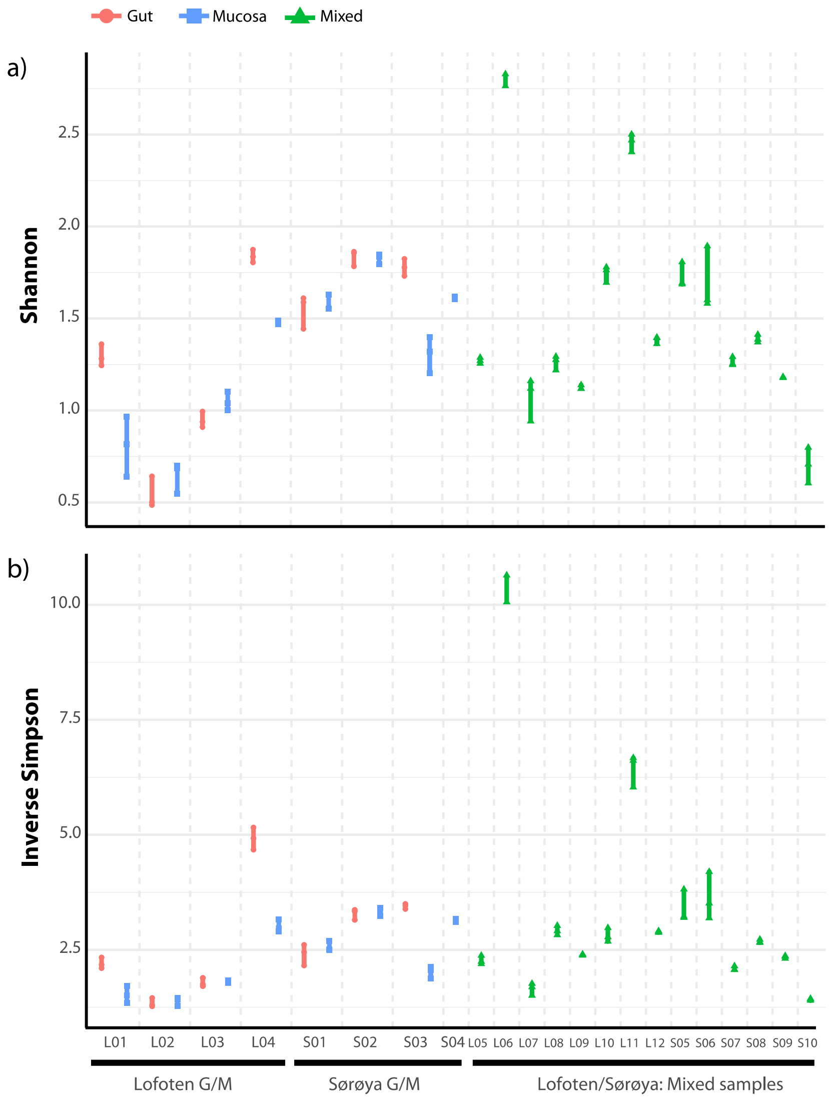


**Figure S3: Shannon (A) and Inverse Simpson (B) diversity in the 22 cod samples.** Each of the 86 replicates is represented by a point. Individuals sampled for both gut (red) and mucosa (blue) on the left half of the figure, individuals sampled for a mix (green) of gut and mucosa on the right half.
